# Devizes Hospital Collection

**Published:** 1897-12-18

**Authors:** 


### DEVIZES HOSPITAL COLLECTION.

In nearly every part of the country there are signs that the working classes not only recognise their indebtedness to the hospitals, but display their sense of responsibility by taking part in their support. As this laudable endeavour is becoming so general, it is all the more to be regretted that at Devizes a retrograde policy has just been adopted. There, for some time, a fund was collected from the friendly societies and workshops by means of penny subscriptions monthly from the members. This fund was devoted to the maintenance of the Cottage Hospital. Quite recently the fund has been closed, owing to the want of co-operation on the part of some of the largest firms—a fact which discouraged the others—and so a bad example has prevailed, and the working men of Devizes have abandoned their self-respecting and public-spirited attitude.

